# Chromatin Targeting Signals, Nucleosome Positioning Mechanism and Non-Coding RNA-Mediated Regulation of the Chromatin Remodeling Complex NoRC

**DOI:** 10.1371/journal.pgen.1004157

**Published:** 2014-03-20

**Authors:** Laura Manelyte, Ralf Strohner, Thomas Gross, Gernot Längst

**Affiliations:** Biochemistry Centre Regensburg (BCR), University of Regensburg, Regensburg, Germany; Indiana University, Howard Hughes Medical Institute, United States of America

## Abstract

Active and repressed ribosomal RNA (rRNA) genes are characterised by specific epigenetic marks and differentially positioned nucleosomes at their promoters. Repression of the rRNA genes requires a non-coding RNA (pRNA) and the presence of the nucleolar remodeling complex (NoRC). ATP-dependent chromatin remodeling enzymes are essential regulators of DNA-dependent processes, and this regulation occurs via the modulation of DNA accessibility in chromatin. We have studied the targeting of NoRC to the rRNA gene promoter; its mechanism of nucleosome positioning, in which a nucleosome is placed over the transcription initiation site; and the functional role of the pRNA. We demonstrate that NoRC is capable of recognising and binding to the nucleosomal rRNA gene promoter on its own and binds with higher affinity the nucleosomes positioned at non-repressive positions. NoRC recognises the promoter nucleosome within a chromatin array and positions the nucleosomes, as observed *in vivo*. NoRC uses the release mechanism of positioning, which is characterised by a reduced affinity for the remodeled substrate. The pRNA specifically binds to NoRC and regulates the enzyme by switching off its ATPase activity. Given the known role of pRNA in tethering NoRC to the rDNA, we propose that pRNA is a key factor that links the chromatin modification activity and scaffolding function of NoRC.

## Author Summary

Tumour cells overexpress ribosomal RNA (rRNA), which is required for ribosome assembly and cell growth. rRNA gene repression is mediated by the chromatin remodeling complex (NoRC) and a non-coding RNA that binds to this enzyme. This study addresses the mechanism of nucleosome positioning by NoRC and the functional role of the non-coding RNA, which is termed pRNA because it corresponds to the promoter sequence. NoRC recognises the promoter nucleosome in a chromatin array with high affinity and uses a release mechanism to position the nucleosome over the transcription initiation site. The pRNA binds specifically to NoRC and inhibits its ATPase activity. We suggest that the RNA retains NoRC at the gene promoter after remodeling, linking its chromatin modification and scaffolding activity to inactive rDNA copies.

## Introduction

Nucleosomes present a major obstacle for the binding of sequence-specific DNA-binding factors, the interaction of positively charged histone tails with DNA and the masking of DNA binding sites that face in towards the histone octamer surface [1], [2]. As a result, all DNA-dependent processes, such as transcription, replication, repair and recombination, are affected by the positioning of nucleosomes on regulatory sites. ATP-dependent chromatin remodeling enzymes, which use energy from ATP hydrolysis to slide, evict or replace histones within nucleosomes, are key modulators of chromatin structure and DNA-dependent processes [3]. Thus, it is of particular importance to reveal their molecular mechanism of nucleosome remodeling, how these enzymes are targeted to their genomic loci and their role in defining nucleosome positions *in vivo* [4]–[10].

In mammalian cells, there are numerous types of remodeling enzymes that associate with different subunits to form remodeling complexes with distinct biological functions. Due to the high combinatorial complexity, it is estimated that several hundred different chromatin remodeling complexes exist in humans. These remodeling enzymes comprise several groups of ATPases classified into the Snf2, ISWI, Mi-2, Chd1, Ino80, ERCC6, ALC1, CHD7, Swr1, RAD54 and Lsh subfamilies [9], [11]. In addition to their diversity, chromatin remodeling enzymes are highly abundant, with approximately one enzyme for every 10 nucleosomes in yeast and human cells [5], [10].

Remodeling enzymes preferentially localise to specific genomic regions, raising the questions of which signals target the enzymes to these locations and what their functions are at these sites [12], [13]. Recently, the continuous sampling model was suggested for the abundant ISWI type remodeling enzymes. According to this model, the enzyme continuously samples all nucleosomes by transiently binding and dissociating without translocation. Only upon introducing additional signals, such as the direct interaction with sequence-specific DNA-binding factors, histone modifications and altered DNA/nucleosome structures, do these nucleosomes become marked as “to be translocated” by converting them to high-affinity substrates [13]. However, there is still a lack of mechanistic proof for the continuous sampling model.

Active rRNA genes cover the promoter-bound nucleosomes from −157 to −2 (relative to the transcription start site), compatible with the binding of the UBF and TIF-IB/SL1 factors required for transcription initiation [8]. On repressed genes, the nucleosome is shifted 24 nt downstream, occluding the TIF-IB binding site [8], [14]. NoRC (nucleolar remodeling complex), which is an ISWI type remodeling enzyme that consists of two subunits, Tip5 (TTFI interacting protein 5) and the Snf2H ATPase, is required to establish the repressed rRNA genes and initiate heterochromatin formation [15], [16]. NoRC is recruited to the rRNA gene by the Transcription Termination Factor-I (TTF-I), which binds upstream of the gene promoter [17]. Recent studies have revealed that NoRC also interacts with the pRNA (promoter-associated RNA), a 150–200 nt long non-coding RNA that is complementary to the rRNA gene promoter sequences and is required for efficient rRNA gene silencing and subsequent DNA methylation [18], [19].

We addressed whether NoRC affects the architecture of the repressed rRNA gene, its mechanism of nucleosome positioning and how the enzyme is targeted to the promoter nucleosome. We demonstrate that, within arrays of nucleosomes, NoRC is capable of recognising the rRNA gene promoter nucleosome with a higher affinity than that for other nucleosomes and that it specifically repositions the nucleosome to the site that was observed *in vivo*. We show that the mechanism of positioning corresponds to a release model of nucleosome positioning, in which NoRC has a reduced affinity for the remodeled substrate. We further studied the role of the pRNA-NoRC interaction and observed that this RNA serves as a negative regulator of NoRC activity, indicating that tight regulation of these enzymes reduces the wasteful turnover of ATP when maintained within chromatin.

## Results

### NoRC requires linker DNA for nucleosome binding and remodeling

**Figure 1:**
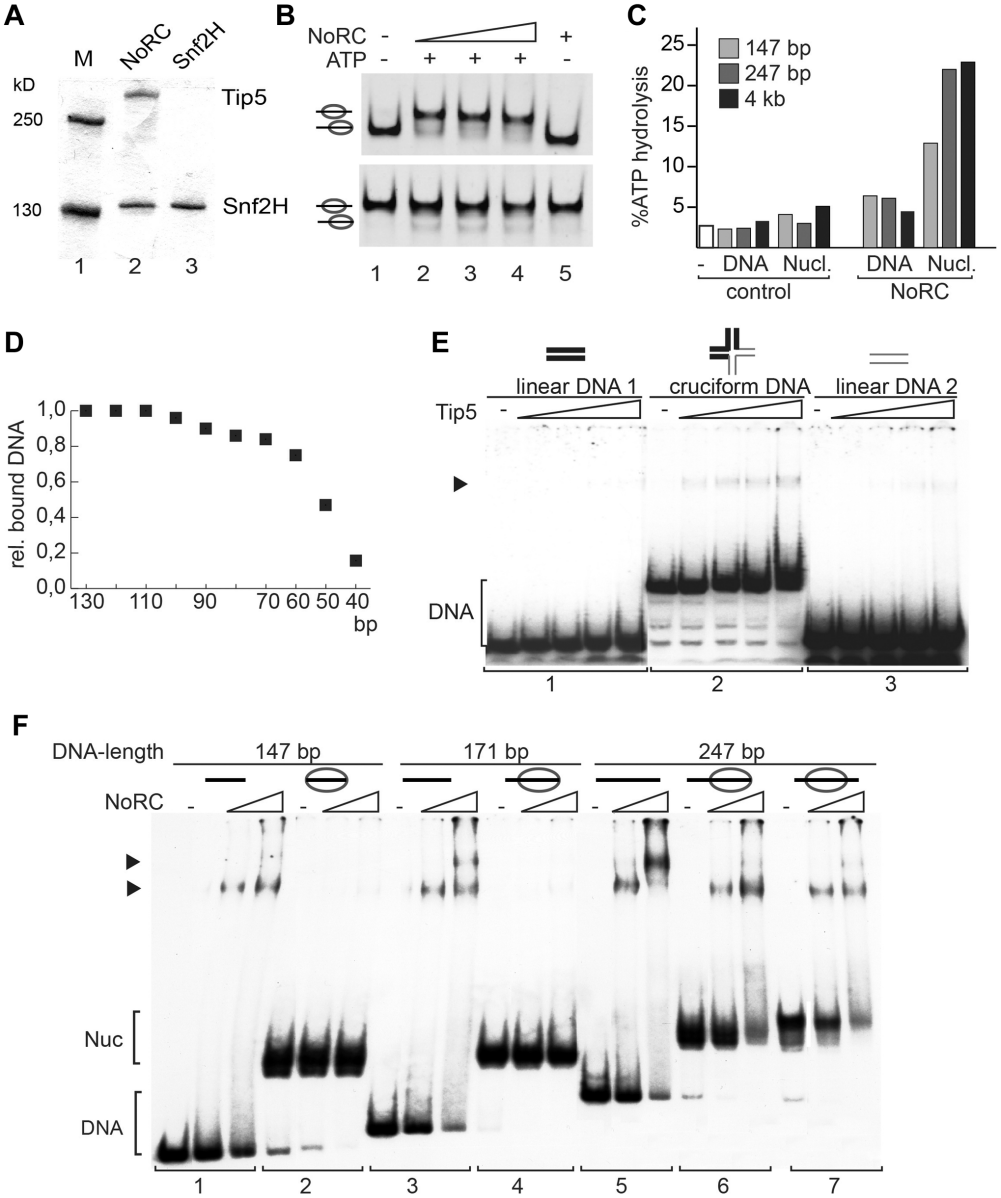
Analysis of NoRC activity and nucleosome binding. (**A**) Purified recombinant NoRC and Snf2H proteins were analysed by SDS-PAGE and Coomassie blue staining. Molecular weight markers are shown on the left and arrows on the right point to the recombinant proteins. (**B**) Remodeling activity of NoRC was tested on nucleosomes reconstituted on 601 DNA [20]. Nucleosomes positioned at the border (upper panel), or at the center of the DNA fragment (lower panel) were incubated with increasing amounts of NoRC and ATP as indicated. After the remodeling reaction the nucleosome positions were analysed on native PAA gels. (**C**) The ATPase activity of NoRC in the presence of DNA and nucleosomes exhibiting different linker lengths. ATP hydrolysis was measured using radioactive ATP as a tracer and the hydrolysed phosphate was separated via thin layer chromatography. Total ATP hydrolysis was quantified and plotted. (**D**) The binding affinity of NoRC to DNA molecules of different length was quantified and plotted. NoRC was incubated with a mixture of DNA molecules of different length and analysed by EMSA. The graph shows a quantification of the relative binding of the individual DNA fragments by NoRC. (**E**) Analysis of Tip5 binding to cruciform DNA. Radioactive labelled cruciform DNA (panel 2) and the two 40 bp DNA controls (panel 1 and 3), which cover the same nucleotide sequence, were incubated with increasing amounts of recombinant Tip5. DNA binding was analysed on native PAA gels. The structure of the annealed oligonucleotides is given on top. (**F**) Binding of NoRC to DNA and nucleosomes with different lengths of linker DNA. Purified nucleosomes, assembled on a 247 bp rDNA fragment, are either positioned at the border or the center of the DNA fragment. The reconstituted nucleosomes contained either no linker DNA (146 bp fragment), ∼25 bp linker DNA (171 bp fragment), ∼50 bp linker DNA (247 bp, middle position) or ∼100 bp linker DNA (end-positioned nucleosome). A scheme of the nucleosomes is shown on the top. Arrowheads indicate the DNA/NoRC or nucleosome/NoRC complexes.

The remodeling complex NoRC, consisting of the Snf2H and Tip5 subunits, was expressed using the baculovirus system and purified to apparent homogeneity (Figure 1A). The activity of NoRC was tested on mononucleosomal substrates reconstituted on the 601 nucleosome positioning sequence in the centre or at the border of the DNA fragment ([20], [21], Figure 1B and S1). The end-positioned nucleosomes were repositioned by NoRC to the central locations in an ATP-dependent remodeling reaction (Figure 1B, upper panel). In contrast, when the nucleosomes were located at the centre of the DNA fragment, only minor ATP-dependent effects were detected (Figure 1B, lower panel). The initial analysis indicated that the recombinant NoRC complex was active but required a specific nucleosomal substrate for its activity.

One of the features of the nucleosomal substrate is the linker DNA. To test whether linker DNA is required for NoRC function, we analysed the ATPase activity of NoRC in the presence of nucleosomal arrays and mononucleosomes with and without linker DNA (Figure 1C and S1D). Interestingly, mononucleosomes lacking linker DNA stimulated the ATPase activity of NoRC significantly less than the linker-containing mononucleosomes or nucleosomal arrays. This experiment suggests that recognition of the nucleoprotein structures in the core nucleosome by NoRC activates its ATPase activity but that linker DNA is required for full stimulation.

Next, to determine the minimal length of DNA required for NoRC binding, we carried out DNA-binding experiments using a mixture of DNA molecules with different lengths (from 10 to 130 bp in 10 bp increments, Figure 1D). Quantification of DNA:NoRC complexes in a competitive assay revealed that the DNA-binding affinity of NoRC strongly decreases with DNA lengths below 60 bp and that the remodeler does not significantly bind to DNA of 40 bp or shorter.

Initial experiments did not demonstrate that Tip5 or NoRC have any sequence-specific DNA binding activity (data not shown). However, NoRC may recognise DNA with a particular structure. Therefore, initial binding of Tip5 to cruciform DNA was analysed. Cruciform DNA and two linear, double-stranded 40 bp DNA fragments (‘DNA sequence controls’) were prepared as described [22]. Increasing amounts of Tip5 were incubated with either the cruciform DNA or the linear DNA and analysed in an electromobility shift assay (EMSA). No binding of Tip5 to either of the linear DNA fragments was visible under the experimental conditions (Figure 1E, panels 1 and 3). In contrast, the incubation of Tip5 with the cruciform DNA resulted in the formation of protein/cruciform DNA complexes (panel 2). The experiment shows preferential binding of NoRC to structured DNA.

To test whether linker DNA is required for a stable interaction of NoRC with the nucleosomes, EMSAs using reconstituted mononucleosomes containing linker DNA of 0 bp (146 bp template), ∼25 bp (171 bp template), ∼50 bp (247 bp template, centrally positioned nucleosome) and ∼100 bp (247 bp template, end-positioned nucleosome) and increasing amounts of NoRC were performed (Figure 1F). NoRC bound with similar affinity to the DNA molecules ranging in length from 146 bp to 247 bp, forming discrete NoRC:DNA complexes as expected from the previous experiment. However, when this DNA was reconstituted into nucleosomes, NoRC failed to form a stable complex with the nucleosomes containing 0 bp and 25 bp of linker DNA but formed discrete NoRC-nucleosome complexes with nucleosomes bearing 50 or 100 bp of linker DNA (Figure 1F). Thus, NoRC has a higher binding affinity for free DNA than nucleosomal cores, which suggests that linker DNA is required for efficient targeting of NoRC to remodeling sites.

### NoRC interacts symmetrically with the nucleosomal edges and the linker DNA

**Figure 2:**
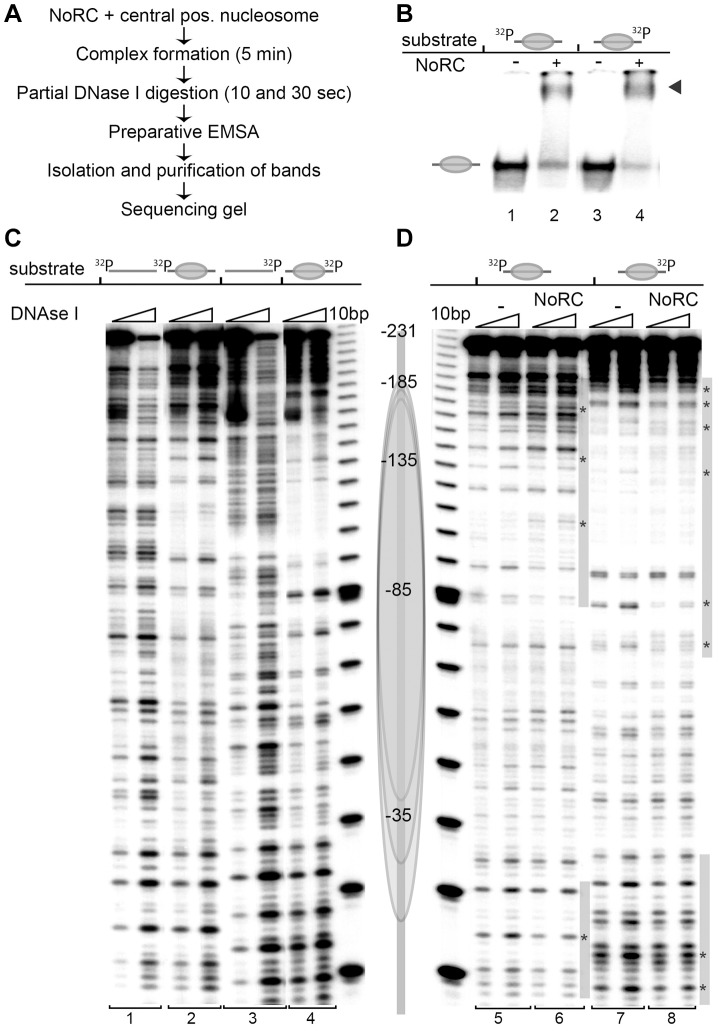
NoRC binds to the entry/exit sites of the nucleosome. (**A**) Overview of the experimental approach. (**B**) Analytical EMSA of the DNase I footprinting reaction. For further analysis of the DNase I digestion pattern the nucleosome and nucleosome/NoRC complexes were isolated from the gel. The arrow indicates the NoRC/nucleosome complexes. (**C**) DNase I footprinting of DNA and centrally positioned nucleosomes. A 247 bp rDNA promoter fragment (−231 to +16 respective to the start site) was radioactively labelled either at the 5′ or 3′ end. The free DNA (bar) and the centrally positioned nucleosome (gray ellipse) were treated with DNase I and after 10 sec and 30 sec the reactions were stopped with EDTA. Nucleosomes and DNA were resolved by EMSA and the bands were isolated. Purified DNA was subsequently analysed on 7% sequencing gels. A scheme of the central positioned nucleosome is shown on the right. (**D**) Recombinant NoRC was incubated with a purified nucleosome positioned at the center of the DNA fragment and partially digested with DNase I (10 and 30 sec). The reaction was stopped by the addition of EDTA and the nucleoprotein complexes were separated by native gel electrophoresis. Nucleosomes and NoRC/nucleosome complexes were isolated, DNA purified and analysed on 7% sequencing gels. The nucleosome position (gray ellipse) and the radioactive end-labeling (^32^P) are indicated. Changes in the digestion pattern upon NoRC treatment are marked with a gray bar, significant changes are highlighted with stars.

To determine the relative orientation of NoRC when bound to the nucleosome, we performed DNase I footprinting experiments. Nucleosomes were reconstituted on the central position of the radioactively end-labelled 247 bp mouse rDNA promoter fragment, a known target site of NoRC [15]. Free DNA, nucleosomes and NoRC-nucleosome complexes were incubated with DNase I, the reaction was stopped by the addition of EDTA and the reaction products were resolved by EMSA (Figure 2A, B). Free DNA, nucleosomes and the corresponding NoRC-nucleosome complexes were gel-purified and further analysed on sequencing gels. When compared to free DNA, DNase I digestion of the nucleosomal DNA resulted in a characteristic cleavage pattern, revealing sites of protection and a repeated pattern of DNase I-sensitive sites with a distance of approximately 10 bp, indicating a nucleosome positioned in the centre of the rDNA fragment (Figure 2C). Because a natural DNA sequence was used in this study, the nucleosome lacked precise positioning and a mixture of rotationally phased nucleosomes broadened the protected region [23]. To avoid the formation of multimeric complexes or template precipitation, NoRC was incubated with the nucleosomal substrates at concentrations that result in 50–70% complex formation. NoRC significantly protected the borders of the nucleosome and the adjacent linker DNA from DNase I digestion (Figure 2D). Our data suggest that the binding of NoRC to the nucleosome is bilateral, interacting with both exit and entry sites of the nucleosome, and confirms that NoRC binds to the linker DNA.

### NoRC determines the nucleosome positions at the rRNA gene promoter

**Figure 3:**
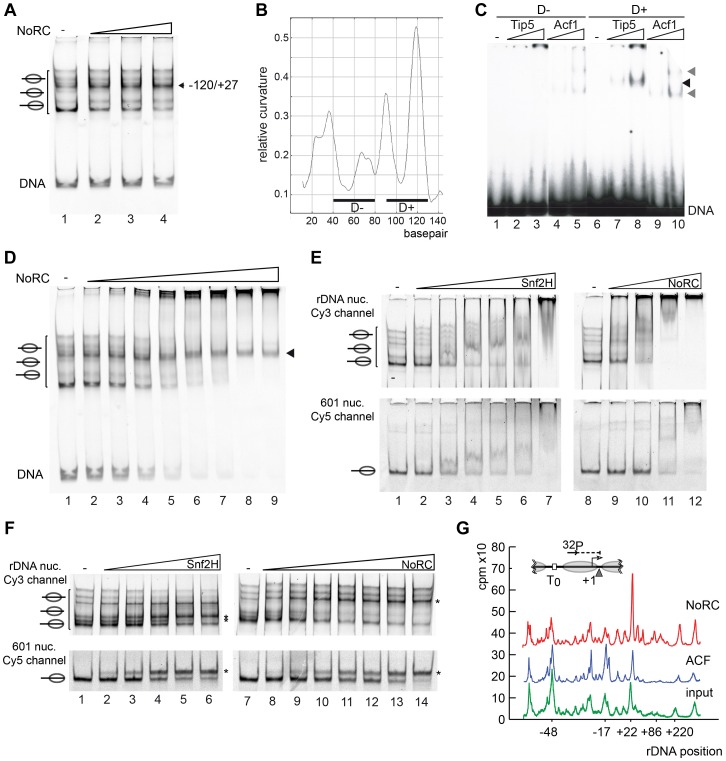
NoRC targeting and remodeling mechanism. (**A**) NoRC repositions nucleosomes reconstituted on the rDNA promoter. Mononucleosomes assembled on the rDNA promoter (DNA from position −190 to +90) were incubated with NoRC (20 to 100 nM) and ATP. The reaction was stopped by addition of competitor DNA and samples were analysed by EMSA. The position of the remodelled nucleosome is shown on the left. (**B**) DNA curvature prediction of the murine rDNA promoter sequence (−231 bp to −95 bp, relative to transcription start site). Curvature was calculated using a DNA curvature prediction program (Bolshoy algorithm/bandit program [26]). The locations and curvatures of the 40 bp DNA fragments used in EMSA are shown: D− (−192 bp to −153 bp) contains nearly no curvature, whereas D+ (−137 bp to −98 bp) oligonucleotide is strongly bent. (**C**) Analysis of Tip5 binding to structured DNA. Increasing amounts of Tip5 and Acf1 were incubated with the radioactively labelled DNA fragments D− and D+ and the complex formation was monitored by EMSA. (**D**) NoRC exhibits a reduced affinity for the remodeled nucleosome. Nucleosomes reconstituted on the rDNA promoter were incubated with increasing NoRC concentrations in the absence of ATP and analysed by EMSA. The arrowhead indicates the nucleosome position −120/+27, the final position of the NoRC dependent remodeling reaction. (**E**) NoRC preferentially binds the rDNA promoter nucleosomes. In the same reaction Cy3-labelled rDNA promoter (upper panel) and Cy5-labelled 601 nucleosomes (lower panel) were incubated with increasing NoRC or Snf2H concentrations in the absence of ATP. Reactions were analysed by EMSA and imaged for the Cy5 and Cy3 channel, respectively. The positions of the nucleosomes are indicated. (**F**) NoRC preferentially remodels the rDNA promoter nucleosomes. Reactions were performed essentially as shown in (**E**), but in the presence of 1 mM ATP. The reactions were stopped with competitor DNA and analysed by EMSA. The respective remodeling reaction is visualized by scanning the Cy3 or the Cy5 channel. The positions of the remodeled nucleosomes are indicated. (**G**) NoRC repositions specifically the promoter nucleosome on an array of nucleosomes. Chromatin was reconstituted on a plasmid DNA containing the rRNA gene promoter and incubated with NoRC or ACF, followed by partial digestion by MNase. The DNA was isolated and analysed by primer extension footprint and denaturing gel electrophoresis. The input chromatin (green), ACF (blue) and NoRC reactions (red) are shown. The relative positions of the peaks to the transcription start site are given.

To examine the ability of NoRC to reposition nucleosomes on its target site, we reconstituted mononucleosomes on a DNA fragment containing the rRNA gene promoter sequence *in vitro* (position −190 to +90, relative to the transcription start site). Nucleosomes reconstituted on the rDNA promoter region occupied multiple positions on the DNA, as demonstrated by native gel electrophoresis (Figure 3A, lane 1). NoRC dependent remodeling establishes a preferential nucleosome position that is located close to the center of the DNA (Figure 3A). This nucleosome position was characterised by Exonuclease III footprinting, showing that it protected the DNA from positions −120 to +27 (Figure S2). This position correlates well with the nucleosome position of the repressed rRNA genes *in vivo* [8]. This suggests that NoRC recognises specific DNA sequences or structures on the nucleoprotein complex that allow site-specific positioning.

A common feature of ribosomal gene promoters is that they lack sequence homology but retain structural similarity and contain intrinsically distorted regions [24]. The relative DNA curvature of the mouse rDNA promoter was calculated with the Bolshoy algorithm using the ‘bandit’ program (Figure 3B, [25], [26]). The mouse rRNA gene promoter contains a region of high local DNA curvature ([25]; at about position −110) that is specifically bound by Tip5 (Figure 3C). This result agrees with the results of the previous experiment, which demonstrated the preferential binding of Tip5 to cruciform DNA (Figure 1E). Thus, these data indicate the specific recognition of structured DNA by the remodeling enzyme, suggesting a potential mechanism for targeting NoRC to the rRNA gene promoter.

### NoRC remodels nucleosomes according to the release model

Two kinetic models were proposed to explain how chromatin remodelers are able to direct the nucleosome to a specific position on DNA [9]. The release model implies that remodelers bind with high affinity to nucleosomes positioned at the “wrong” sites and remodel the nucleosome until it reaches the final (correct) position. The nucleosome at the final position exhibits the lowest affinity for the remodeling enzyme and is thus the worst substrate for the remodeling enzyme. In contrast, the arrest model postulates that the nucleosome exhibits a much higher affinity for the remodeling enzyme at the final position, locking it on the nucleosome and reducing the catalytic conversion rate [9], [27]. To assign one of the kinetic models for a particular remodeler, the binding and remodeling of nucleosomes must be compared. Thus, we compared the differential binding affinities of NoRC to the individual nucleosome positions by EMSA. The incubation of rDNA −190/+90 reconstituted into nucleosomes with increasing concentrations of NoRC resulted in a stepwise binding of the different nucleosome species (Figure 3D). Free DNA and most of the nucleosomes were bound with similar affinities and retarded in the gel. However, the nucleosome occupying the −120/+27 position bound with the lowest affinity. This nucleosome position is the final position of the NoRC-dependent remodeling reaction (Figure 3A), revealing that NoRC has the lowest binding affinity for the “remodeled” nucleosome, therefore suggesting that NoRC remodels nucleosomes according to the release model.

### Tip5 targets NoRC to the rDNA promoter

Differential local binding affinities are required to position nucleosomes on DNA. However, on a more global scale, differential binding affinities could also serve to target the remodeling enzymes to specific genes and regulatory regions. To test how NoRC and Snf2H select their remodeling targets, we used competitive binding and remodeling assays. Nucleosomes were reconstituted on a fluorescently labelled rRNA gene promoter fragment (Cy5 labelled) and the 601 nucleosome positioning sequence ([20], Cy3 labelled). Nucleosomes were mixed and binding or remodeling reactions were performed with increasing amounts of remodelers. Snf2H bound with similar affinity to both nucleosome substrates, and remodeled them with similar efficiency (Figure 3E, F). In contrast, NoRC showed preferential binding to the nucleosomes reconstituted on the rRNA gene promoter, preferentially binding the DNA and nucleosomes at lower NoRC concentrations when compared to the 601 substrate (Figure 3E, lanes 8 to 12). Binding with higher affinity was mirrored in the remodeling assay where NoRC was remodeling the rRNA gene promoter nucleosomes prior to the 601 nucleosomes (Figure 3F and Figure S3).

### NoRC selectively remodels the promoter nucleosome within a nucleosomal array

As cellular nucleosomes are arranged in arrays, we tested whether NoRC is also capable of selectively recognising and repositioning the rRNA gene promoter nucleosome within nucleosomal arrays. Chromatin was reconstituted using the salt dialysis method on a circular DNA containing the rRNA gene promoter and incubated with NoRC or ACF in the presence of ATP. A partial MNase digestion of the nucleosomal DNA was performed and analysed in a primer extension reaction (Figure 3G). ACF did not qualitatively change the distribution of the nucleosomes within the analysed region of the rRNA gene promoter. However, NoRC induced a specific relocalisation of the promoter nucleosomes, placing the 3′ end of the nucleosome at position +22. NoRC-dependent nucleosome positioning at +22 perfectly corresponds to the cellular nucleosomal configuration of the repressed rRNA gene [8]. The 5 bp discrepancy between the mononucleosome remodeling and array remodeling assay could arise from internucleosomal interactions that influence the remodeling outcome. Our data strongly support the hypothesis that nucleosome remodeling complexes determine nucleosome positioning *in vivo*, thereby directly affecting gene expression.

Previous studies have revealed a specific interaction between TTF-I and NoRC, suggesting that TTF-I recruits NoRC to the rRNA gene promoter [15], [17]. The results described here reveal an additional targeting signal, encoded by the high affinity of NoRC for nucleosomes positioned at “wrong” sites of the rDNA promoter. TTF-I improves the efficiency of NoRC recruitment to the rRNA gene promoter without affecting the outcome of the NoRC-dependent nucleosome remodeling reaction (Figure S4).

### pRNA switches off the remodeling activity of NoRC

**Figure 4:**
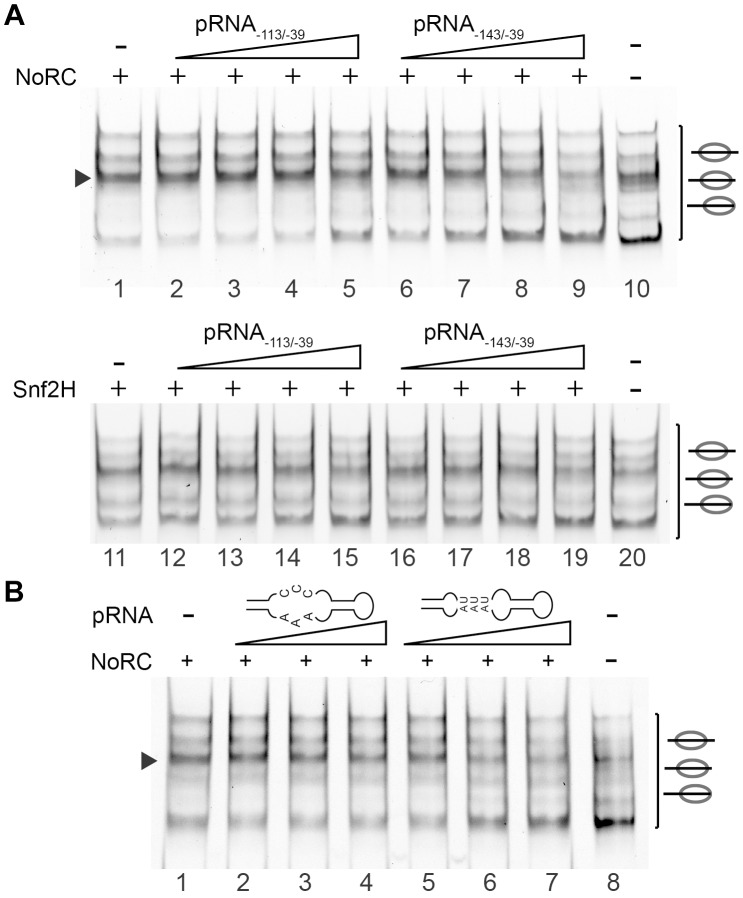
pRNA inhibits the activity of NoRC. (**A**) Nucleosomes assembled on the −190 to +90 rDNA DNA fragment were incubated with NoRC or Snf2H, ATP and increasing concentrations of pRNA_−143/−39_ and pRNA_−113/−39_ (5 to 200 nM). The remodeling reactions were analysed by EMSA. The arrowhead indicates the nucleosome at the −120/+27 position. (**B**) NoRC specifically recognizes the hairpin-loop structure of the pRNA. Mononucleosomes assembled on −190 to +90 rDNA promoter region were incubated with NoRC and increasing concentrations of pRNA_−127/−39_ (20 to 80 nM) or the mutated pRNA missing the hairpin-loop structure in the presence of ATP. Remodeling reactions were stopped after 45 min and analysed by EMSA. The arrowhead indicates the nucleosome at the −120/+27 position.

Recent studies have demonstrated that NoRC binds to a non-coding RNA, which is initiated upstream of the rRNA gene promoter and contains promoter sequences in the sense orientation. It was suggested that promoter RNA (pRNA) is required to tether NoRC to inactive rRNA genes, where it establishes repressive epigenetic marks [18], [19], [28]. We studied two pRNA constructs that exhibit strong and weak binding affinities for Tip5, pRNA_−143/−39_ and pRNA_−113/−39_, respectively [19]. pRNAs were generated by *in vitro* transcription, re-natured and added to the remodeling reactions (Figure 4). First, the presence of the pRNAs did not influence the nucleosome positioning behaviour of NoRC. Second, we observed specific inhibition of the NoRC-dependent remodeling reaction with increasing levels of pRNA_−143/−39_ (Figure 4A). In contrast, Snf2H was similarly inhibited by both pRNAs, suggesting that the Tip5 subunit determines RNA-binding specificity and activity. Moreover, NoRC recognises the secondary structure of the pRNA, as inhibition of its nucleosome-remodeling activity was lost when the stem-loop structure was mutated (Figure 4B). We identified a regulatory role of the pRNA, demonstrating that the non-coding RNA serves as an inhibitor of the remodeling enzyme.

**Figure 5:**
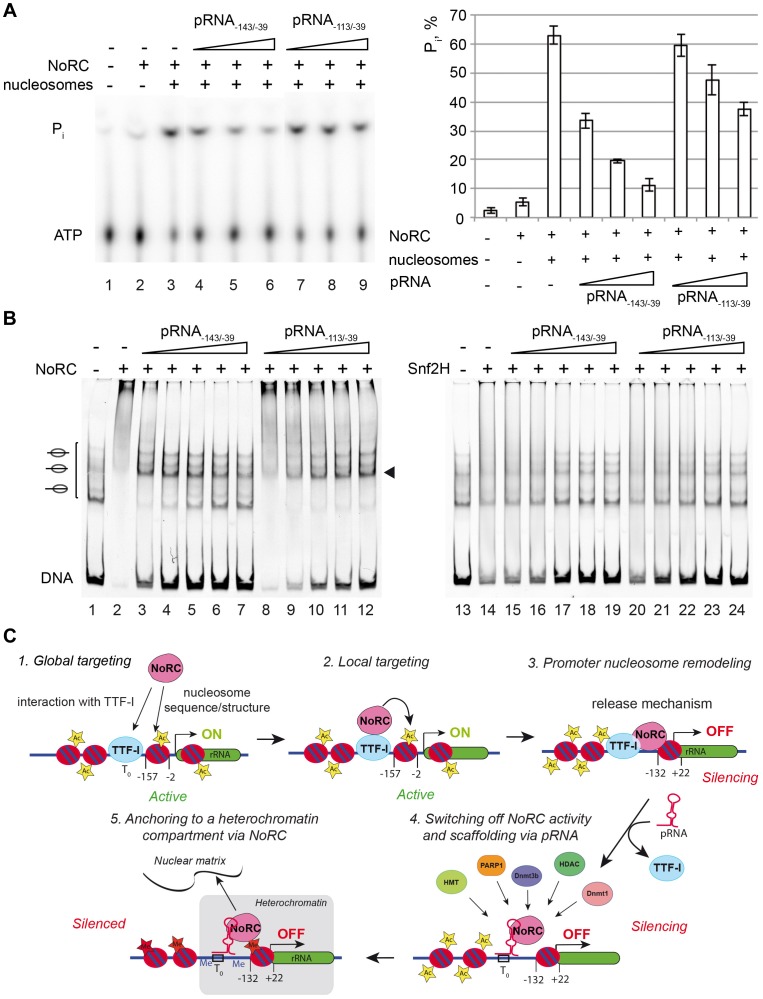
Nucleosomes and pRNA compete for the binding of NoRC. (**A**) ATPase assay. NoRC was incubated with the indicated pRNAs and radioactive ATP as a tracer. Hydrolysed phosphate was separated via thin layer chromatography and analysed on a PhosphoImager. The quantification of three independent reactions is plotted. Error bars show the standard deviations. (**B**) Competitive binding assays using NoRC, pRNA and nucleosomes. Nucleosomes assembled on the rDNA promoter (−190 to +90) (lane 1) were incubated with NoRC (lanes 2 to 12), resulting in quantitative complex formation (lanes 2 and 8). These complexes were incubated with increasing concentrations of pRNA as indicated and analysed by EMSA. The nucleosome occupying the position −120/+27 is indicated. Lanes 13 to 24 shows the experiment, but performed with Snf2H. (**C**) Model describing the putative roles of NoRC and pRNA in rRNA gene silencing.

To gain more insight into the inhibitory mechanism of pRNA, we investigated the effect of pRNA on NoRC ATPase activity. The incubation of NoRC with an increasing amount of DNA or pRNA only modestly stimulated the NoRC ATPase activity (Figure S5), whereas the presence of nucleosomes considerably accelerated ATP/ADP exchange. The incubation of NoRC with nucleosomes and increasing amounts of pRNA_−143/−39_ or pRNA_−113/−39_ resulted in a RNA concentration-dependent inhibition of the ATPase activity (Figure 5A). As in the remodeling reaction, pRNA_−143/−39_ inhibited the NoRC-dependent ATPase activity more efficiently than pRNA_−113/−39_, confirming the higher binding affinity of the remodeling complex for this RNA and explaining the inhibition of the nucleosome remodeling reaction.

To reveal the mode of RNA-dependent inhibition, we studied the binding of NoRC to nucleosomes in the presence of RNA (Figure 5B). A competitive EMSA revealed that the pRNA competes with nucleosomes for NoRC binding, indicating that only exclusive NoRC:pRNA or NoRC:nucleosomes complexes exist. Again, competition of nucleosomes from the NoRC:nucleosome complex required less pRNA_−143/−39_ than pRNA_−113/−39_, indicating the higher binding affinity of pRNA_−143/−39_ for NoRC (Figure 5B). Both RNA species competed similarly with Snf2H, pointing to the specific role of Tip5 in NoRC (lanes 13 to 24). In summary, our data demonstrate that pRNA competes with nucleosomes for NoRC binding and therefore directly interferes with its ATPase activity and the nucleosome remodeling reaction.

## Discussion

NoRC is an ISWI type remodeling enzyme that requires linker DNA for nucleosome binding and efficient activation of its ATPase activity and remodeling. The complex recognises structured and non-structured DNA with a minimal length of 30 bp, and the same length of linker DNA is required for stable interactions with nucleosomes. Our data suggest that the most stable interactions are formed with the linker DNA rather than the nucleosome core, as we were not able to detect interactions between NoRC and the nucleosome core in electromobility shift assays. Reduced binding affinities to the nucleosome core potentially explain the reduced ATPase activity observed with NoRC using nucleosome cores. However, binding to the linker DNA and the orientation of the complex with respect to the nucleosome core are not random, as specific interactions with the DNA entry/exit sites of the nucleosome were visible in DNase I footprinting experiments. NoRC was specifically aligned adjacent to the nucleosome, giving rise to symmetrical DNase I protected and enhanced cleavage sites, a pattern reminiscent of ACF binding to nucleosomes [23], [29].

### Recognition and remodeling of the rRNA gene promoter

Ribosomal genes present an ideal model system for studying the dynamics and mechanism of chromatin remodeling, as the epigenetic marks, the chromatin structure of the active and repressed genes and the factors involved are well characterised. Active rRNA genes contain a nucleosome covering the gene promoter from positions −157 to −2, allowing the binding of UBF and TIF-IB/SL1 to their recognition sites at the nucleosomal borders [8]. In contrast, repressed genes have a nucleosome covering the positions from −132 to +22 relative to the transcription start site, masking the binding site of TIF-IB. The repression of rRNA genes is intimately linked with the recruitment of NoRC, which induces nucleosome remodeling, gene repression and the acquisition of heterochromatic marks [16]. We show that the activity of NoRC is sufficient for recognition of the promoter structure and nucleosome positioning *in vivo*. Nucleosomal arrays are required to establish the cellular nucleosome positioning pattern, suggesting that internucleosomal interactions influence the activity of remodeling enzymes. Our results are in good agreement with data demonstrating that ISWI machines are molecular rulers and potentially act in the context of di-nucleosomes [30], [31]. Although NoRC does not serve as a sequence-independent spacing factor, it is capable of recognising sequence features of the rRNA gene promoter, which serve as positioning signals.

Several studies have demonstrated the importance of positioned nucleosomes in the genome [32]. However, irrespective of the ability of many sequences to position nucleosomes *in vitro* they fail to do so *in vivo* [33], [34], suggesting that there are additional mechanisms that structure chromatin. We show that NoRC positions nucleosomes according to the release mechanism [9], [13]. The enzyme binds with high affinity to nucleosomes positioned at “wrong” sites, which is the recruitment signal. The remodeling reaction is highly processive, with ACF moving a nucleosome for approximately 200 bp without leaving the nucleosomal substrate [35]. After initiation of the remodeling reaction, the endpoint of the translocation reaction is determined by a reduced affinity of the remodeler for the nucleosome at this site. As any remodeler with distinct binding affinities to nucleosomes at different but close positions on DNA could position nucleosomes, we suggest that chromatin remodeling enzymes serve to organise chromatin structure with respect to the underlying DNA sequence. The concentration and composition of the remodeling enzymes in combination with the specific targeting of those complexes to chromatin would determine a specific chromatin architecture and specify accessible regulatory sequences that determine the activity of DNA-dependent processes. We suggest that the combinatorial aspect of remodeling enzymes and complex constitution may determine cell types and their responses to the environment.

There are a multitude of signals targeting remodeling enzymes to specific genomic regions, including direct recruitment by proteins, protein modifications, histone variants, coding and non-coding RNAs, as well as nucleosomes at “wrong” positions [13]. The continuous sampling model for chromatin remodeling enzymes suggests that high concentrations of remodeling enzymes and low binding affinities towards the non-signalling nucleosomes allow for efficient screening of the genome for signals that attract remodeling enzymes [10]. Here, we provide evidence for the continuous sampling mechanism of NoRC, where the remodeling enzyme selectively remodels the promoter nucleosome within an array of nucleosomes. Differential binding affinities guide the remodeling enzyme to these sites of action. However, on the genomic scale additional targeting signals help to further increase the local concentration of the remodeling enzymes at their sites of action. In the case of NoRC, interaction with TTF-I directly recruits NoRC and thereby improves the efficiency of the remodeling reaction, but does not influence the remodeling outcome [14], [17].

### Effect of pRNA on NoRC-dependent remodeling

Previous studies have shown that the TAM domain in the Tip5 subunit interacts with pRNA and that this interaction is a prerequisite for maintaining NoRC in the nucleolus [18]. We show that pRNA competes with nucleosomes for NoRC binding, specifically inhibiting its ATPase activity. Therefore, we suggest that a ternary complex consisting of NoRC, nucleosomes and RNA does not exist, despite the fact that NoRC contains several DNA/nucleosome-binding domains and an RNA-binding TAM domain [36].

We suggest, that the pRNA serves three functions (Figure 5C). First, after replacing TTF-I at the rRNA gene promoter, it serves to maintain NoRC localisation at the promoter. Due to the release mechanism of nucleosome positioning, NoRC has a low affinity for the remodeled chromatin structure and most likely would dissociate from the promoter. Given, that Grummt and colleagues have shown that the 5′-end of the pRNA forms a triplex with the T_0_ site at the promoter region and that the 3′-end interacts with Tip5, we propose a tethering function for the pRNA. Switching off the ATPase activity of NoRC ensures that the nucleosome is stably maintained in the OFF position and that the enzyme does not waste ATP. The pRNA and NoRC recruit DNA methyltransferases, histone deacetylases and histone methyltransferases to silence the rRNA genes [37]–[39] and recruit the silenced genes to the heterochromatin environment of the nuclear matrix [36].

## Materials and Methods

### Proteins

The proteins were expressed in SF21 cells. N-terminally His tagged Snf2H with or without Tip5 was purified via Ni-NTA (Qiagen) chromatography. Flag tagged Snf2H and Acf1 were purified using M2 beads (Sigma) [14].

### DNA and RNA preparation

Murine rRNA gene promoter fragments of 146 bp (−231 to −86; positions relative to the transcription start site), 171 bp (−231 to −61), 247 bp (−231 to +16) and 280 bp (−190 to +90) were amplified by PCR from a plasmid containing the genomic DNA isolated from the NIH3T3 cell line (genbank access #KC202874.1). To radioactively label the DNA fragments, α-^32^P dCTP was added to the PCR reaction mix. The 601 DNA and the pRNA were prepared by PCR as described [19], [40]. PCR products were used for nucleosome assembly reactions as described [23].

### Nucleosome assembly

Nucleosomes were assembled according to Rhodes and Laskey using the salt gradient dialysis technique [41]. A typical assembly reaction (50 µl) contained 5 µg DNA, varying amounts of histone octamer, 200 ng BSA/ml, and 250 ng competitor DNA in high salt buffer (10 mM Tris, pH 7.6, 2 M NaCl, 1 mM EDTA, 0.05% NP-40, 2 mM β-mercaptoethanol). The salt was continuously reduced to 200 mM NaCl during 16–20 h. The quality of the assembly reaction was analysed on a 5% PAA gel in 0.4× TBE followed by ethidium bromide staining. Nucleosomes reconstituted on the 247 bp rDNA promoter fragment display two distinct positions that can be separated by native gel electrophoresis [21].

### Nucleosome remodeling assay

Nucleosome mobility was assayed as described [42]. Briefly, reactions contained 4 nM Cy5 labelled DNA reconstituted into nucleosomes, 1 mM ATP, 100 ng/µl BSA, 1 mM DTT, 70 mM imidazole in Ex80 buffer (20 mM Tris pH 7.6, 80 mM KCl, 1.5 mM MgCl_2_, 0.5 mM EGTA, 1 mM β-mercaptoethanol, 10% glycerol, 200 ng/µl BSA) and recombinant remodeling enzymes. Nucleosomes were incubated with NoRC for 45 min at 30°C. The reactions were stopped by the addition of 1700 ng CMV14 plasmid DNA and incubated for 15 min on ice. The nucleosome positions were analysed by electrophoresis on 5% PAA gels in 0.4× TBE and fluorescence scanning.

### DNA and nucleosome binding assays

Tip5 binding to cruciform DNA was performed as described [22]. NoRC binding to the DNA and nucleosomes was studied by electromobility shift assays (EMSA). The substrates used in the assay were either radioactively or fluorescently labelled as indicated in the legends. Reactions were performed in Ex80 buffer and the indicated amounts of NoRC. Reactions were incubated for 45 min at 30°C and then analysed by native PAGE. Competitive titration experiments were performed using identical reaction conditions, containing 25 nM NoRC, 4 nM fluorescently labelled mononucleosomes and the indicated amounts of the indicated pRNA constructs. The reactions were analysed on 5% polyacrylamide gels in 0.4× TBE and subsequent fluorescence scanning.

### DNaseI footprinting assay

NoRC/nucleosome and nucleosome DNase I footprinting experiments were performed as described [29]. Essentially, radioactively end-labelled DNA was reconstituted into nucleosomes and incubated with NoRC using the same experimental conditions as in the remodeling reactions. DNase I digestions were stopped by the addition of EDTA to a final concentration of 5 mM. The complexes were resolved on native PAA gels and the DNA, nucleosome and NoRC/nucleosome complexes were excised from the gel. DNA was purified and analysed on 7% sequencing gels. Mapping nucleosomal boundaries on nucleosomal arrays before, or after remodelling with NoRC or ACF was performed as described [14].

### ATPase assay

An ATPase reaction contained 150 ng of DNA or chromatin in 10 µl of Ex75 buffer, 10 µM ATP and γ^32^P-ATP (0.1 µl; 3000Ci/mmol, Hartmann Analytic), the indicated amounts of pRNA_−143/−39_ or pRNA_−113/−39_ and 10 units RNasin. The reactions were initiated by the addition of the remodeling enzyme and incubated for 60 min at 30°C. Aliquots of 1 µl were spotted on thin layer cellulose chromatography plates (Merck) and air-dried. The hydrolyzed phosphate was separated from unreacted ATP by thin layer chromatography in 0.5 M LiCl/acetic acid buffer. The plates were dried at 65°C for 5 min and exposed to Phospho Imager plates (FujiFilm BAS-1500). ATP and hydrolyzed phosphate spots were quantified using the Multigauge software (Fuji). The percentage of hydrolyzed ATP was calculated according to the following equation: P_i_/(ATP+P_i_)×100%, where P_i_: amount of hydrolyzed radioactive phosphate; ATP: amount of left γ^32^P-ATP.

### Exonuclease III mapping of nucleosome boundaries

Nucleosome positioning on the Cy5 5′ end-labelled mouse rDNA fragment (from positions −190 to +90 relative to the transcription start site) was determined with Exo III mapping. Reactions were carried out in an initial volume of 50 µl with 30 nM nucleosomes and 2 U/µl of Exo III (NEB) in 10 mM Tris, 90 mM KCl,1 mM MgCl_2_, and 1 mM DTT at 16°C. At different time points 7 µl of the reaction mix were removed and the reaction was stopped by the addition of EDTA (final concentration of 50 mM). Proteins were digested with Proteinase K after the addition of SDS to a final concentration of 1% and the DNA was subsequently purified by ethanol precipitation. DNA samples were analysed on 6% sequencing gels. The DNA ladder was prepared with the DNA Cycle Sequencing Kit (Jena Bioscience) using a Cy5 labelled oligonucleotide and the mouse rDNA promoter fragment (−190 to +90), with either ddTTP or ddCTP in the reaction mix. Results were imaged with a FLA-5000 imager (Fujifilm). As control, we carried out Exo III digestions with naked DNA in order to discriminate nucleosome positions from exonuclease pause sites on free DNA. To map NoRC dependent positions a remodeling reaction was performed prior to Exo III analysis. Remodeling was performed with 7.4 ng/µl of NoRC and Cy5 labelled nucleosomes in the presence or absence of 1 mM ATP for 60 min at 30°C. The reaction was stopped with competitor plasmid DNA and used for native gel analysis and Exo III footprinting.

## Supporting Information

Figure S1 Characterization of NoRC complex. (**A**) Purified recombinant Snf2h, Tip5 and NoRC proteins were analysed by SDS-PAGE and Coomassie blue staining. (**B**) The ATPase activity of Snf2H, Tip5 and NoRC in the presence or absence of a nucleosome array. ATP hydrolysis was measured using radioactive ATP as a tracer and the hydrolysed phosphate was separated via thin layer chromatography. Quantification of hydrolysed ATP is shown. (**C**) Remodeling activity of NoRC was tested on nucleosomes reconstituted on Hsp70 DNA [9]. Mononucleosomes were incubated with increasing concentrations of NoRC and ATP as indicated. Nucleosome remodeling reactions were analysed on native PAA gels. (**D**) The ATPase activity of NoRC in the presence of 100 or 300 ng of nucleosomes with or without linker DNA was analysed. ATP hydrolysis was measured using radioactive ATP as a tracer and the hydrolysed phosphate was separated via thin layer chromatography. Quantification of ATP hydrolysis is given.
(TIF)

Figure S2 Analysis of nucleosome positions by Exonuclease III mapping. (**A**) Nucleosome assembly on the Cy5 labelled rDNA promoter. Reconstituted mononucleosomes were analysed on a native 6% PAA gel. (**B**) Exo III digestion of DNA and nucleosomes was performed for 0 to 20 min. The purified DNA was analysed on a 6% sequencing gel followed by fluorescence scanning. Specific nucleosomal stop sites are indicated with asterisks. (**C**) Schematic summary of the identified nucleosomal positions on the rDNA promoter fragment determined in (**B**). (**D**) PAA gel showing the NoRC remodeling reaction used for the Exo III analysis. Cy5 labelled nucleosomes were incubated with NoRC in the presence or absence of ATP as indicated. Changes in nucleosome positioning were analysed on native PAA gels. (**E**) Determination of the NoRC dependent nucleosome position. Exo III boundaries of nucleosomes, or nucleosomes in the presence of NoRC, with or without ATP, as indicated were determined as described in (**B**). The NoRC dependent nucleosome position are given. The Sequencing ladder of the T and C reaction is shown on the left.
(TIF)

Figure S3 Competitive remodeling of the rDNA promoter nucleosomes and the 601 nucleosome by NoRC. (**A**) In the same reaction Cy5-labelledrDNA promoter and Cy3-labelled 601 nucleosomes were incubated with increasing concentrations of Snf2H in the presence of 1 mM ATP. The reactions were stopped with competitor DNA, the remodeling reactions were analysed by EMSA and imaged for the Cy5 and Cy3 channel, respectively. (**B**) The quantitation of the Snf2H dependent remodeling data is given. (**C,D**) Same experimental setup as described in (**A, B**), but the remodeling enzyme NoRC was used.
(TIF)

Figure S4 TTF-I increases the efficiency of NoRC dependent remodeling on the rRNA gene promoter. A nucleosomal array reconstituted by the salt dialysis method was incubated with NoRC, or NoRC and TTF-I and ATP for 30 min. The remodeling reaction was partially digested with MNase and the DNA was purified. Primer extension reactions using a radioactive labelled primer was performed on the purified DNA. The products were analysed by denaturing gel electrophoresis and quantified with a PhosphorImager. The traces for the input chromatin and the chromatin after remodeling with NoRC, or NoRC and TTF-I are shown in green, red and black. The position of the peaks relative to the transcription start site of the rRNA gene are given.
(TIF)

Figure S5 Effect of RNA, DNA and nucleosomes on the ATPase activity of NoRC. (**A**) NoRC (190 nM) was incubated with increasing concentrations of the DNA, nucleosomes and RNA substrates (15 nM, 30 nM, 60 nM). ATP hydrolysis was measured for 1 h at 30°C using radioactive ATP as a tracer. Hydrolysed phosphates were separated by thin layer chromatography. (**B**) Quantification of the ATP hydrolysis of three independent experiments like shown in (**A**). The standard deviation is given.
(TIF)

Text S1 Supplementary Materials and Methods.
(DOCX)
