# Supplementary figures and images for: Chromatin Targeting Signals, Nucleosome Positioning Mechanism and Non-Coding RNA-Mediated Regulation of the Chromatin Remodeling Complex NoRC
Source: PLoS Genet. 2014 Mar 20;10(3):e1004157. doi: 10.1371/journal.pgen.1004157 (PMC3961174; doi:10.1371/journal.pgen.1004157)

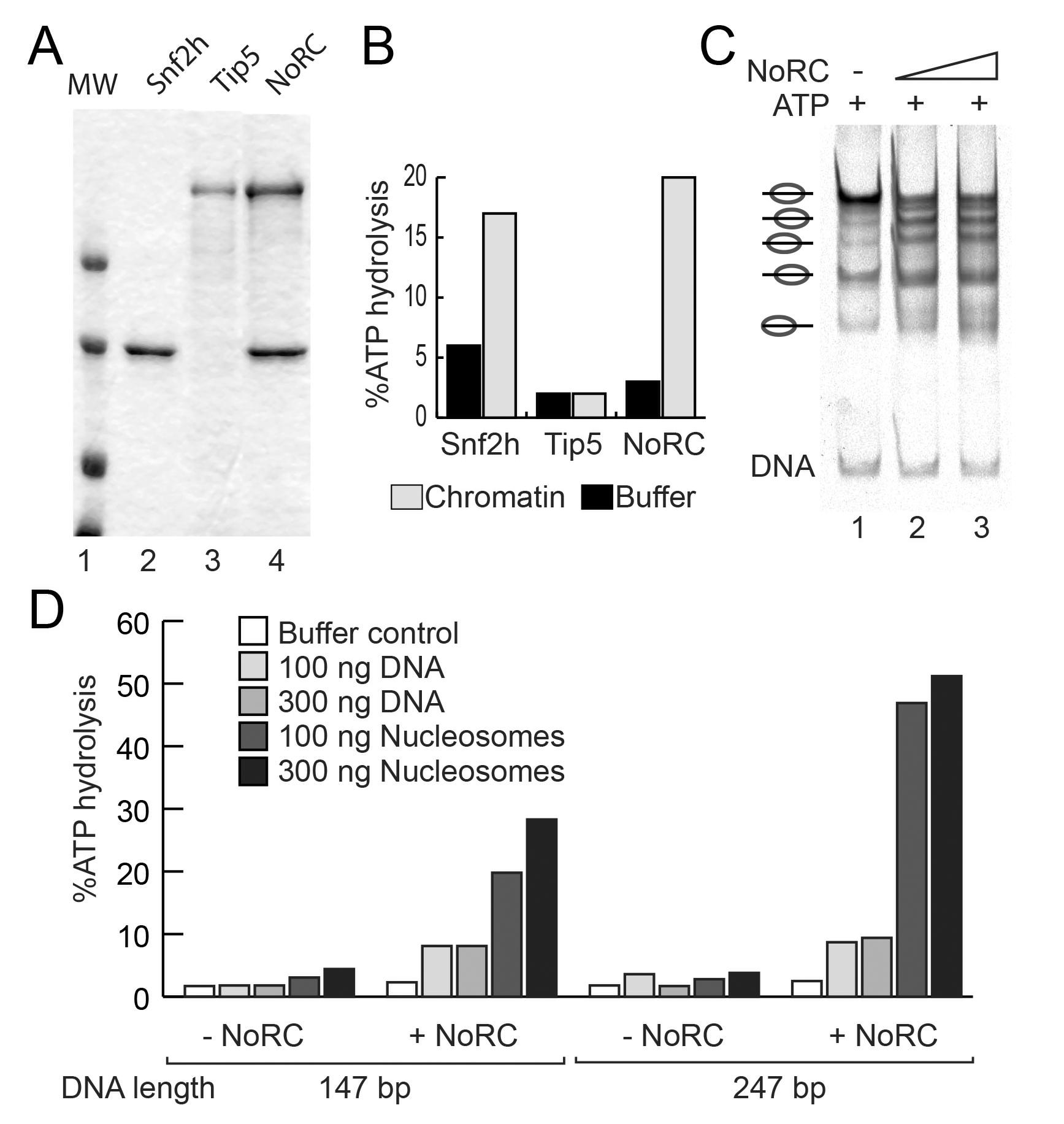

Supplement: Figure S1 — Characterization of NoRC complex. (A) Purified recombinant Snf2h, Tip5 and NoRC proteins were analysed by SDS-PAGE and Coomassie blue staining. (B) The ATPase activity of Snf2H, Tip5 and NoRC in the presence or absence of a nucleosome array. ATP hydrolysis was measured using radioactive ATP as a tracer and the hydrolysed phosphate was separated via thin layer chromatography. Quantification of hydrolysed ATP is shown. (C) Remodeling activity of NoRC was tested on nucleosomes reconstituted on Hsp70 DNA [9]. Mononucleosomes were incubated with increasing concentrations of NoRC and ATP as indicated. Nucleosome remodeling reactions were analysed on native PAA gels. (D) The ATPase activity of NoRC in the presence of 100 or 300 ng of nucleosomes with or without linker DNA was analysed. ATP hydrolysis was measured using radioactive ATP as a tracer and the hydrolysed phosphate was separated via thin layer chromatography. Quantification of ATP hydrolysis is given. (TIF) [file pgen.1004157.s001.tif]

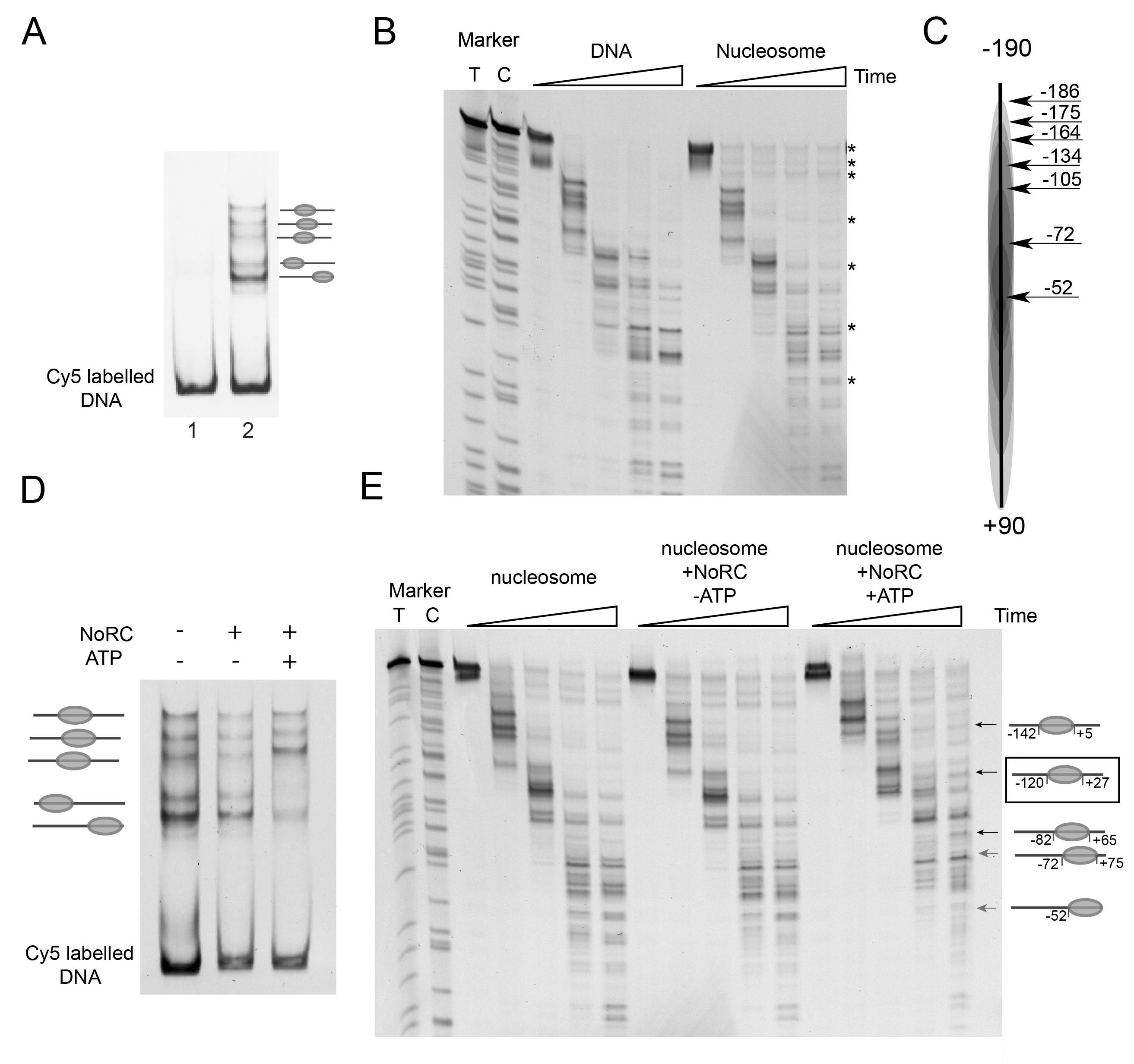

Supplement: Figure S2 — Analysis of nucleosome positions by Exonuclease III mapping. (A) Nucleosome assembly on the Cy5 labelled rDNA promoter. Reconstituted mononucleosomes were analysed on a native 6% PAA gel. (B) Exo III digestion of DNA and nucleosomes was performed for 0 to 20 min. The purified DNA was analysed on a 6% sequencing gel followed by fluorescence scanning. Specific nucleosomal stop sites are indicated with asterisks. (C) Schematic summary of the identified nucleosomal positions on the rDNA promoter fragment determined in (B). (D) PAA gel showing the NoRC remodeling reaction used for the Exo III analysis. Cy5 labelled nucleosomes were incubated with NoRC in the presence or absence of ATP as indicated. Changes in nucleosome positioning were analysed on native PAA gels. (E) Determination of the NoRC dependent nucleosome position. Exo III boundaries of nucleosomes, or nucleosomes in the presence of NoRC, with or without ATP, as indicated were determined as described in (B). The NoRC dependent nucleosome position are given. The Sequencing ladder of the T and C reaction is shown on the left. (TIF) [file pgen.1004157.s002.tif]

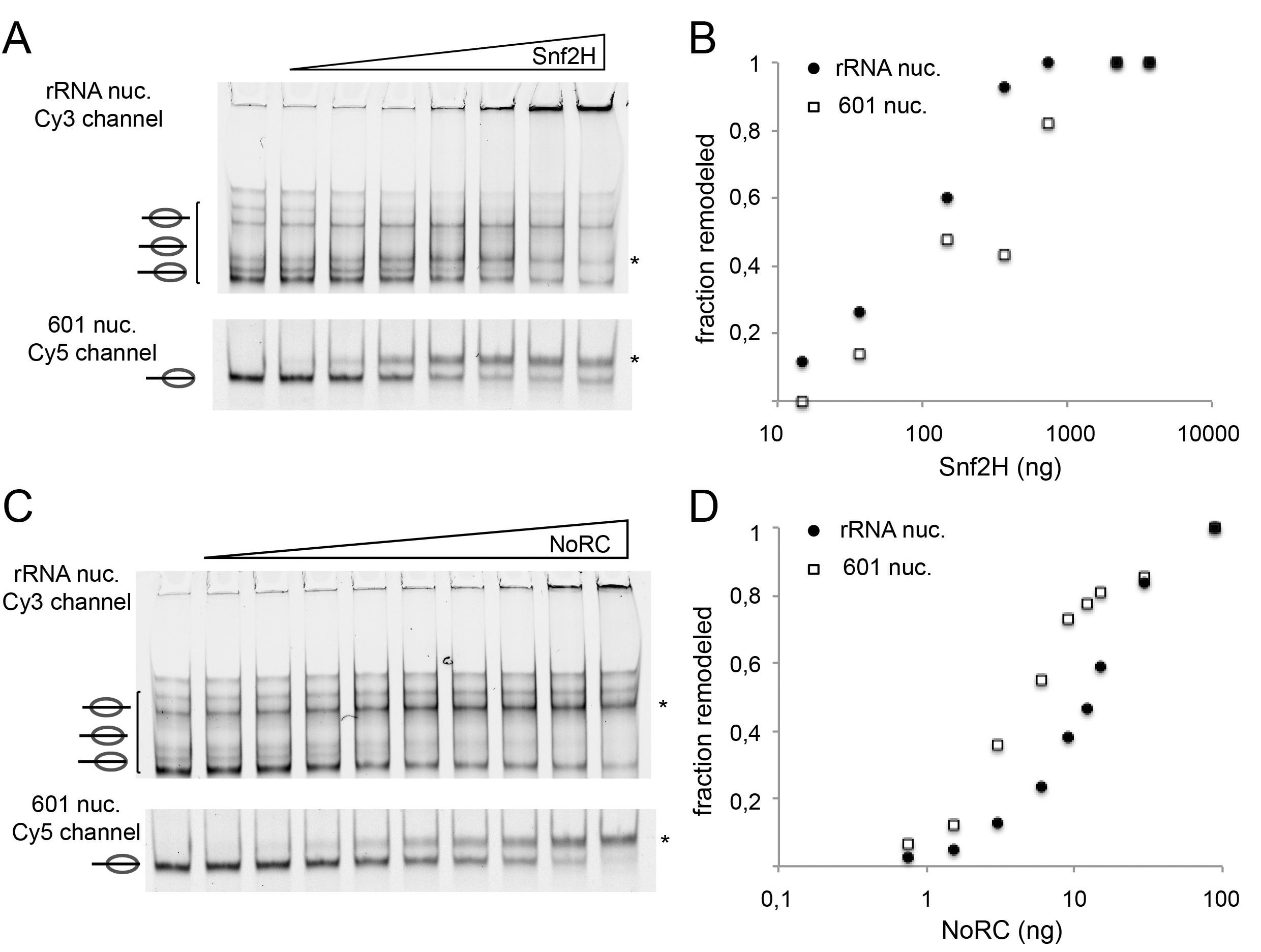

Supplement: Figure S3 — Competitive remodeling of the rDNA promoter nucleosomes and the 601 nucleosome by NoRC. (A) In the same reaction Cy5-labelledrDNA promoter and Cy3-labelled 601 nucleosomes were incubated with increasing concentrations of Snf2H in the presence of 1 mM ATP. The reactions were stopped with competitor DNA, the remodeling reactions were analysed by EMSA and imaged for the Cy5 and Cy3 channel, respectively. (B) The quantitation of the Snf2H dependent remodeling data is given. (C,D) Same experimental setup as described in (A, B), but the remodeling enzyme NoRC was used. (TIF) [file pgen.1004157.s003.tif]

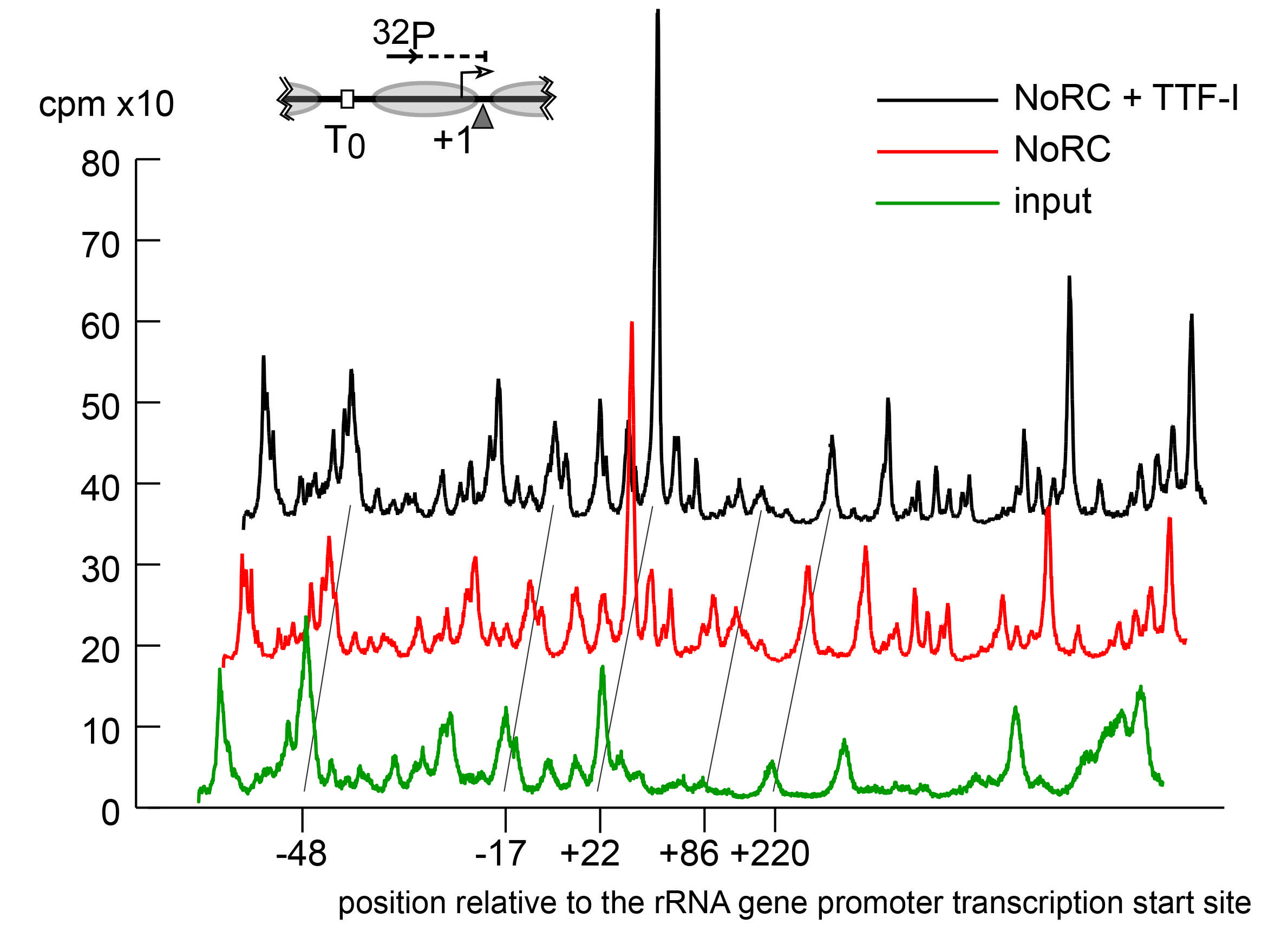

Supplement: Figure S4 — TTF-I increases the efficiency of NoRC dependent remodeling on the rRNA gene promoter. A nucleosomal array reconstituted by the salt dialysis method was incubated with NoRC, or NoRC and TTF-I and ATP for 30 min. The remodeling reaction was partially digested with MNase and the DNA was purified. Primer extension reactions using a radioactive labelled primer was performed on the purified DNA. The products were analysed by denaturing gel electrophoresis and quantified with a PhosphorImager. The traces for the input chromatin and the chromatin after remodeling with NoRC, or NoRC and TTF-I are shown in green, red and black. The position of the peaks relative to the transcription start site of the rRNA gene are given. (TIF) [file pgen.1004157.s004.tif]

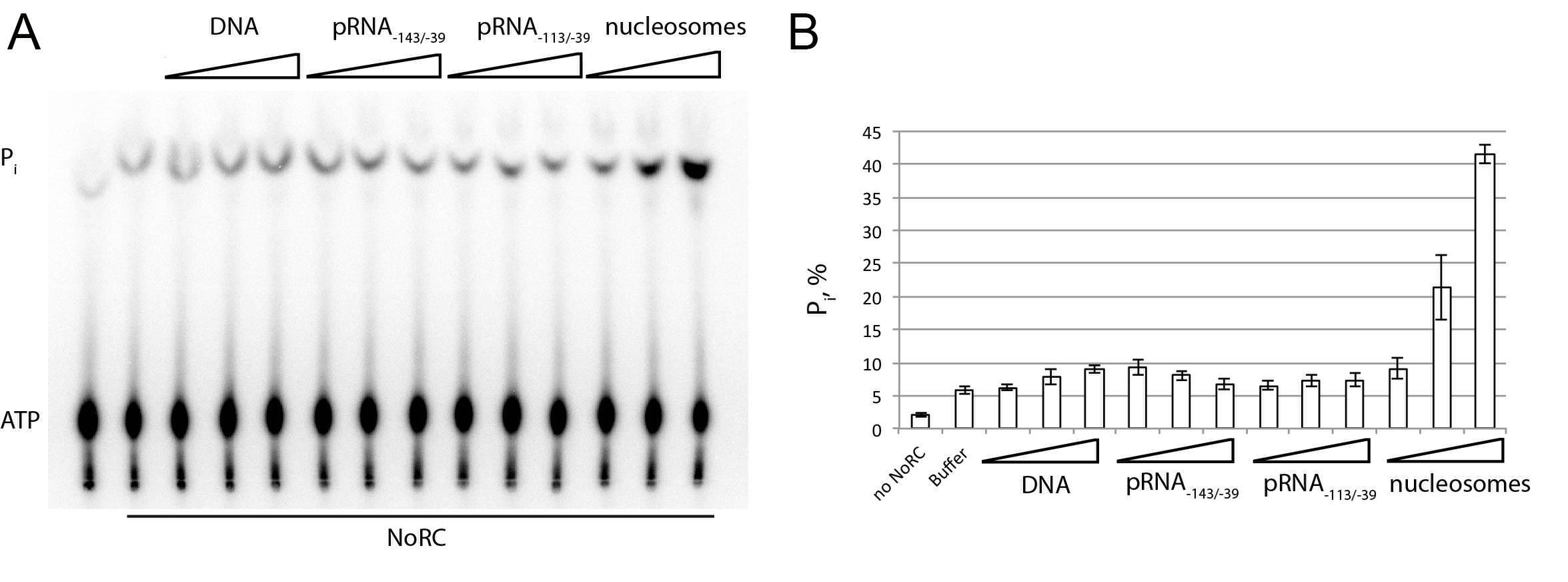

Supplement: Figure S5 — Effect of RNA, DNA and nucleosomes on the ATPase activity of NoRC. (A) NoRC (190 nM) was incubated with increasing concentrations of the DNA, nucleosomes and RNA substrates (15 nM, 30 nM, 60 nM). ATP hydrolysis was measured for 1 h at 30°C using radioactive ATP as a tracer. Hydrolysed phosphates were separated by thin layer chromatography. (B) Quantification of the ATP hydrolysis of three independent experiments like shown in (A). The standard deviation is given. (TIF) [file pgen.1004157.s005.tif]
