# Supplementary material for: Chromatin Targeting Signals, Nucleosome Positioning Mechanism and Non-Coding RNA-Mediated Regulation of the Chromatin Remodeling Complex NoRC
Source: PLoS Genet. 2014 Mar 20;10(3):e1004157. doi: 10.1371/journal.pgen.1004157 (PMC3961174; doi:10.1371/journal.pgen.1004157)
Supplement: Text S1 — Supplementary Materials and Methods. (DOCX) [file pgen.1004157.s006.docx]

**Chromatin targeting signals, nucleosome positioning mechanism and non-coding RNA-mediated regulation of the NoRC chromatin remodeling complex**

Laura Manelyte, Ralf Strohner, Thomas Gross and Gernot Längst^*^

Biochemistry Centre Regensburg (BCR), University of Regensburg, Universitätsstr. 31, D-93053 Regensburg, Germany.

* Contact: gernot.laengst@ur.de, phone: +49 941 943 2849, fax: +49 941 943 2474

**File contains:**

**Supplementary Material and Methods**

**Supplementary Materials and Method**

**DNA templates**

280 bp murine rDNA fragment (-190 to +90) was amplified from genomic DNA isolated from NIH3T3 cell line (genbank access #KC202874.1) via PCR using primers (mDNA_90_rev_Cy5-GAATAGGCTGGACAAGCAAAACAGCC and mDNA_-190_for TATCAGTTCTCCGGGTTGTCAGGTC).

The 146 bp, 170 bp and 247 bp murine rDNA fragments were amplified using primers 146 bp (LP7 / LP-86), 171 bp (LP7 / LP-61) and 247 bp (LP7 / LP2):

LP7: GAAAGCTATGGGCGCGGTTTT

LP-86: CCTGGAAGTCATACCTGGG

LP-61: GAAAGTGACAGGCCACAGA

LP2: GGACAGCGTGTCAGTACCTA

**Exonuclease III mapping of nucleosome boundaries**

Nuclesome positioning on the Cy5 5’ end-labelled mouse rDNA fragment (from positions -190 to +90 relative to the transcription start site) was determined with Exo III mapping. Reactions were carried out in an initial volume of 50 µl with 30 nM nucleosomes and 2 U/µl of Exo III (NEB) in 10 mM Tris, 90 mM KCl,1 mM MgCl_2_, and 1 mM DTT at 16°C. At different time points 7 µl of the reaction mix were removed and the reaction was stopped by the addition of EDTA (final concentration of 50 mM). Proteins were digested with Proteinase K in after addition of SDS to a final concentration of 1% and the DNA was subsequently purified by ethanol precipitation. Purified DNA samples were analysed on 6% sequencing gels. The DNA ladder was prepared with the DNA Cycle Sequencing Kit (Jena Bioscience) using a Cy5 labeled oligonucleotide and the mouse rDNA promoter DNA (-190 to +90), with either ddTTP or ddCTP in the reaction mix. Results were imaged with a FLA-5000 imager (Fujifilm). As control, we carried out Exo III digestions with naked DNA in order to discriminate nucleosome positions from exonuclease pause sites on free DNA. To map NoRC dependent positions a remodeling reaction was performed prior to Exo III analysis. Remodeling was performed with 7.4 ng/µl of NoRC, Cy5 labeled nucleosomes in the presence or absence of 1 mM ATP for 60 min at 30°C. The reaction was stopped with competitor plasmid DNA and used for native gel analysis and Exo III footprinting.

**References**

1. Rippe K, Schrader A, Riede P, Strohner R, Lehmann E, et al. (2007) DNA sequence- and conformation-directed positioning of nucleosomes by chromatin-remodeling complexes. Proc Natl Acad Sci USA 104: 15635–15640. doi:10.1073/pnas.0702430104.
